# Determination of the Isotopic Composition of Indium by MC-ICP-MS Using an Improved Measurement Model for the Gravimetric Isotope Mixture Method

**DOI:** 10.1021/acs.analchem.5c00878

**Published:** 2025-08-15

**Authors:** Juris Meija, Kenny Nadeau, Brad Methven, Lu Yang

**Affiliations:** 103209 National Research Council Canada, Metrology Research Centre, 1200 Montreal Road, Ottawa, Ontario K1A 0R6, Canada

## Abstract

In this study, we report the first independent and primary isotope ratio measurement of indium by MC-ICP-MS, utilizing gravimetric isotope mixtures of two near-pure enriched indium isotopes. Consistent with previous findings, we observe that the traditional one-mixture-at-a-time calibration approach can introduce a strong dependence of the resulting isotope ratio correction factors on the composition of the mixtures. Our analysis suggests that this is due to biases inherent in measuring the isotopic composition of pure isotopic materials. To address this issue, we propose an improved calibration strategy that omits the use of isotope ratios measured in the two near-pure indium isotopes, at the expense of measuring additional isotope mixtures. The revised calibration approach yields an indium isotope ratio *n*(^113^In)/*n*(^115^In) = 0.044 655 ± 0.000 009 (95% confidence level) for the high-purity indium isotopic reference material (NRC HIIN-1). This result is in agreement with our 2010 result (0.044 72 ± 0.000 19, 95% confidence level), obtained using the regression method with NIST SRM978a silver as a calibrator, while showing an improvement in measurement uncertainty by an order of magnitude.

## Graphical abstract

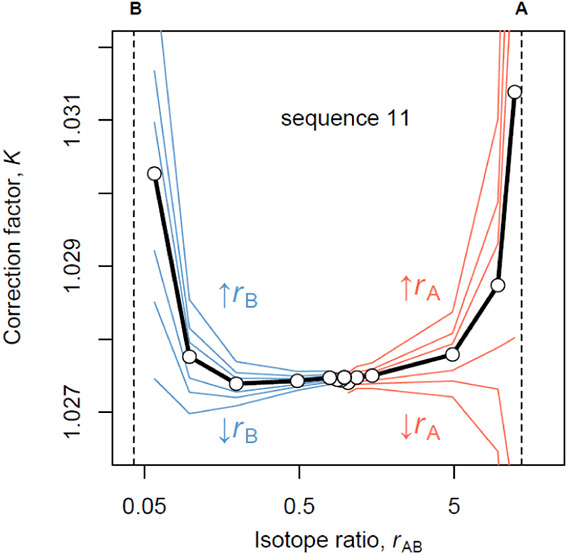

## Introduction

Indium is a critical element in modern technology, primarily due to its application in liquid crystal display (LCD) production.^1^ It exists in the natural environment at low abundances, typically less than 0.1 μg/g in the upper Earth’s crust, and reaches percent levels in certain ore-forming minerals.^2–5^ With the economically most important source for indium being sphalerite, the majority of global indium production is derived from zinc processing pipelines.^2–5^ Despite its technological importance, the geochemical behavior of indium is not well studied, and only a limited number of studies have focused on indium isotope ratio measurements.^6–10^ Indium is a volatile and chalcophile element, and its isotopic variations offer valuable insights into core-mantle differentiation and volatile depletion in planets. Additionally, variations in indium isotope ratios can be used to trace anthropogenic sources of indium contamination in the environment. Therefore, high-precision indium isotope ratio measurements are crucial for supporting these applications.

High-precision isotopic analysis plays an increasingly critical role in science, as variations in the isotopic compositions of elements provide valuable insights into geological, biological, and chemical processes. The isotopic analysis of stable isotopes of heavy elements is typically performed using thermal ionization mass spectrometry (TIMS) or multicollector inductively coupled plasma mass spectrometry (MC-ICP-MS). Regardless of the mass spectrometry technique employed, high-precision isotope ratio measurements require corrections for instrumental isotopic fractionation, which in turn calls for isotopic standards. Such standards either provide reliable isotopic compositions or serve as easily accessible reference materials against which other materials can be compared.

In this vein, the aim of this study is to achieve, for the first time, a calibrated high-precision indium isotope ratio measurement that does not rely on any other isotopic standard. This is accomplished using gravimetric isotope mixtures of near-pure indium isotopes. In order to make use of these measurements, we prepared and characterized a high-purity indium isotopic reference material (NRC HIIN-1), providing a solid basis for future isotopic studies of indium.

## Experimental Section

### Instrumentation

A Neptune Plus (Thermo Fisher Scientific, Bremen, Germany) MC-ICP-MS instrument was used for indium isotope ratio measurements at low mass resolution mode. Our MC-ICP-MS is equipped with nine Faraday cups, a quartz dual cyclonic spray chamber, and a PFA self-aspirating nebulizer (Elemental Scientific, Omaha, NE, USA) operating at a flow rate of 50 μL min^–1^. The instrument was tuned daily for high sensitivity while maintaining a flat-top peak shape (square) and stable signals. The gain calibration of the Faraday cups was performed weekly to ensure normalization of their efficiencies. Rotating amplifiers were chosen during the measurements, as they effectively minimize all calibration biases associated with the amplifiers and improve measurement precision. Typical operating conditions are presented in Table S1.

### Reagents and Solutions

High-purity nitric acid was obtained from a subboiling distillation system (Milestone Inc., Shelton, CT, USA) using reagent-grade HNO_3_ (Fisher Scientific, Ottawa, ON, Canada). Deionized water (DIW, 18.5 MΩ cm) was obtained from a Milli-Q ion exchange system (Sigma-Aldrich, Oakville, ON, Canada). All labware was soaked in 5% HNO_3_ solution for several days and then rinsed with DIW prior to use.

### Sample Preparation

The indium isotopic reference material, NRC HIIN-1, was prepared from a high-purity indium metal wire of 2 mm diameter (Amalgamet Canada; Toronto, ON, Canada). The wire was cut into 1.1 g pieces (30 mm in length) using a wire electrical discharge technique at the NRC and bottled in 4 mL glass vials filled with argon.

Near-pure indium-113 isotope in metallic form (Trace Sciences Inc.; Richmond Hill, ON, Canada) was employed in this study. It had a nominal isotopic abundance, *x*(^113^In) = 0.935 mol/mol, and we designate it here as material A. Since natural indium predominantly consists of indium-115, *x*(^115^In) = 0.945 mol/mol, one unit of NRC HIIN-1, designated herein as material B, was used as indium-115 for calibration. The elemental impurities in both materials A and B were assessed using glow discharge mass spectrometry (GDMS) at the NRC. Afterward, both materials were cleaned with 10% HNO_3_, rinsed with water, and dried in a class-100 fume hood before their quantitative dissolution in high-purity HNO_3_. Gravimetric dilution was performed to yield mass fractions *w*(In, A0) = 3997.45 mg kg^–1^ and *w*(In, B0) = 9988.13 mg kg^–1^, where buoyancy corrections were applied during the calculations. Serial gravimetric dilutions of these two stock solutions with 2% HNO_3_ produced two sets of 1 μg kg^–1^ working solutions of materials A and B.

Antimony was used as the internal standard for indium isotope delta ratio measurements. A 2000 mg kg^–1^ stock solution of antimony was prepared by quantitative dissolution of high-purity antimony metal (0.99999 g/g purity; 5N Plus, Saint-Laurent, QC, Canada) in a few mL of high-purity HNO_3_ and HF, followed by dilution with deionized water. A working standard solution of antimony (100 mg kg^–1^) was prepared by dilution of the stock with 2% HNO_3_.

A total of 15 stock solutions (labeled In_1_ to In_15_) of NRC HIIN-1 were prepared from 15 individual units of HIIN-1 metal rods, each weighing approximately 1.1 g. The rods were cleaned with 10% HNO_3_, rinsed with water, and dried under argon in a class-100 fume hood. The pins were weighted and then individually dissolved in small amounts (few mL) of warm high-purity HNO_3_ and then diluted with deionized water to result in a nominal mass fraction of *w*(In) = 10000 mg kg^–1^. A mixed stock solution (labeled HIIN-mix) was prepared by blending equal aliquots (2 g each) of In_1_ to In_15_ solutions. A serial dilution of this stock was used to prepare a HIIN-mix working solution with *w*(In) ≈1 mg kg^–1^.

Although a minimum of three mixtures are sufficient to derive the isotope ratio calibration factor, *K*, without using pure materials A and B^11^, a total of 14 mixtures were gravimetrically prepared by mixing the solutions A1 and B1. This was done to investigate the reliability of *K* values across a range of isotopic compositions of the gravimetric mixtures. The isotopic composition of these mixtures spanned from ^113^In/^115^In = 0.06 to 13. Two sets of such mixtures were prepared by weighing appropriate amounts of solutions of A1 and B1 or A2 and B2, followed by dilution with 2% HNO_3_ to yield a final mass fraction of 0.5 mg kg^–1^ of indium Table S2). To ensure consistency, individual solutions of A1, B1, and HIIN-mix were all prepared in a similar manner to match the mass fraction of indium across all samples. A 2% HNO_3_ solution was used as the blank.

### Measurement Procedure

The two sets of prepared test solutions were analyzed using MC-ICP-MS in the following measurement sequence: blank, AB1, A1, B1, HIIN-mix, HIIN-mix, HIIN-mix, AB2, AB1, blank, AB3, AB4, AB5, AB6, AB7, AB8, AB1, blank, AB9, AB10, AB11, AB12, AB13, AB14, AB1, blank, HIIN-mix, HIIN-mix, HIIN-mix, and AB1. This measurement sequence was repeated, with minor deviations, eight times for each set of mixtures, as shown in Table S2. Measurements were conducted over the period from July 2022 to January 2023.

A 90-s washout with 2% HNO_3_ was performed between each sample measurement. An additional 5 min wash was conducted before and after analyzing the pure materials. It was found to effectively reduce the indium signals to blank levels. Additionally, intensities of indium isotopes, measured at peak mass in the first bracketing blank solution (2% HNO_3_), were subtracted from the analytical signals of the bracketed samples. Indium isotope signals were also continuously monitored between the measurements with 2% HNO_3_ blank solution to ensure that they returned to blank levels before proceeding with the next sample. Since this method involves a sequential measurement of all pure enriched materials and mixtures, efforts were made to match both the total mass fraction of indium and the sample matrix (nitric acid level) across all solutions. To ensure stable signals, the instrument was conditioned for at least 1 h prior to each measurement sequence. The solutions AB1–1 and AB1–2 (both having ^113^In/^115^In ≈ 1) were repeatedly measured throughout each measurement sequence to evaluate the isotope ratio drift. Half of the measurements displayed isotope ratio drift below 200 ppm (Figure 1). In all cases, linear piecewise interpolation was used to perform the drift correction.

**Figure 1:**
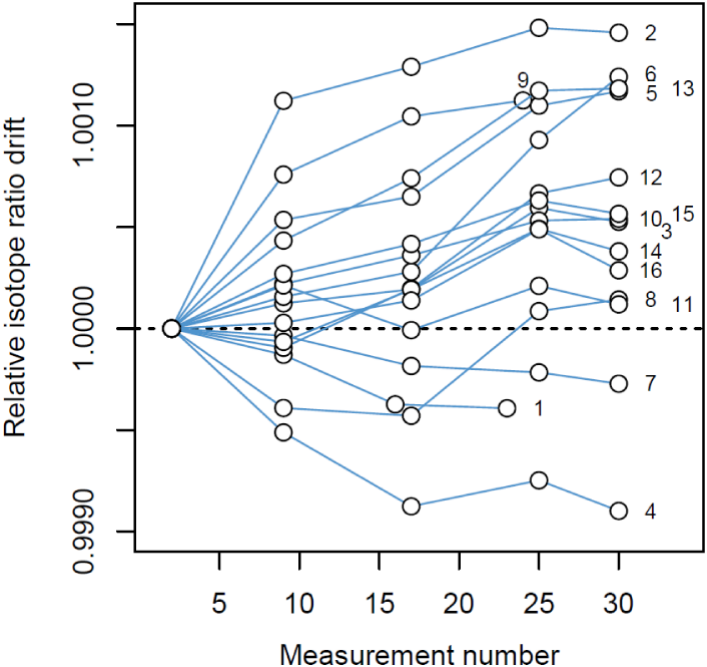
Indium isotope ratio (^113^In/^115^In) drifts, evaluated from the measurements of samples AB1–1 and AB1–2, occurred during each of the 16 measurement sequences. Linear piecewise interpolation was used to correct for isotope ratio drifts within each measurement sequence.

### Spectral Interferences

Both the indium-113 (material A) and the natural indium (material B) are high-purity metals, and potential polyatomic interferences from ^75^As^38^Ar^+^, ^73^Ge^40^Ar^+^, ^77^Se^36^Ar^+^, ^97^Mo^16^O^+^, ^75^As^40^Ar^+^, ^81^Br^34^S^+^, ^99^Ru^16^O^+^, and ^97^ Mo^18^O^+^ on indium isotopes ^113^In and ^115^In are negligible, because As, Ge, Mo, S, Ru, and Mo are present at levels below 90 ng g^–1^ in these two materials (Table S3). However, the Cd content is 14 mg kg^–1^ in material B, and the Sn content exceeds 2 mg kg^–1^ in both materials. To investigate possible isobaric interferences from ^113^Cd and ^115^Sn on the two indium isotopes, interference-free isotopes ^111^Cd and ^117^Sn were monitored. It was found that the signals for ^111^Cd and ^117^Sn isotopes in both solutions A and B (at a mass fraction of 0.5 mg kg^–1^ of indium) were indistinguishable from the signals obtained from the 2% HNO_3_ blank, confirming that the isobaric interferences of ^113^Cd and ^115^Sn are negligible.

### Evaluation of HIIN-1 Homogeneity

To evaluate the homogeneity of indium isotope ratios in the NRC HIIN-1 reference material, aliquots from the 15 stock solutions of HIIN-1, made by dissolving 15 individual units of HIIN-1, were each diluted and spiked with the stock solution of antimony in 2% HNO_3_ to yield *w*(In) = 0.8 mg kg^–1^ and *w*(Sb) = 1.5 mg kg^–1^. Relative isotope ratios (isotope deltas) of these 15 indium standard solutions (In_1_ to In_15_) were measured against the mixture solution (HIIN-mix) using the combined standard-sample bracketing method with antimony as the internal standard (C-SSBIN).^12^

## Results and Discussion

### Chemical Purity of Indium Materials

The success of isotope ratio calibration using the gravimetric isotope mixture model depends critically on the chemical purity of the near-pure isotopes of the element. High-purity metal rods of indium isotopes were sourced, and their chemical purity was assessed using GDMS at the NRC.^13,14^ Elemental impurities of A and B materials were determined by GDMS using measurement models and methods with traceability to the SI through a network of CRMs.^13,14^ For indium-113 (Material A), the primary impurities were identified as copper, platinum, lead, and cadmium (Table S3). For the natural indium (material B), tin and lead constituted the major impurities. Purity values of *w*(In, A) = 0.999 768(70) kg kg^–1^ and *w*(In, B) = 0.999 940(7) kg kg^–1^ (standard uncertainties are quoted in parentheses) were obtained for materials A and B by subtracting the sum of all individual elemental impurities listed in Table S3.

Note that the stock solutions A1 and B1 were prepared by weighing out precleaned metallic indium materials of ^113^In (A) and ^115^In (B). The chemical purity of indium in these materials, as assessed by GDMS, was taken into account in order to obtain the mass fraction of indium, as well as buoyancy corrections.

### Isotope Ratio Correction (Traditional Model)

Calibration of isotope ratios can be achieved by preparing mixtures of isotopically enriched materials.^12^ The relationship between the observed (*r*) and true isotope ratios (*R*) is modeled with the correction factor *K*, whereby *R* = *Kr*. For indium, the correction factor *K* can be obtained using the traditional approach, where the isotope ratio ^113^In/^115^In is measured in three materials: material A (*r*_A_), material B (*r*_B_), and their mixture (*r*_AB_). To derive the isotope ratio correction factor (*K*), the following expression is commonly used:^11^

1 $$K=\frac{m_{a,115}}{m_{a,113}}\cdot \frac{m_{A(\mathrm{AB})}\cdot \left(r_{\mathrm{AB}}-r_{A}\right)+m_{B(\mathrm{AB})}\cdot \left(r_{\mathrm{AB}}-r_{B}\right)}{m_{A(\mathrm{AB})}\cdot r_{B}\cdot \left(r_{A}-r_{\mathrm{AB}}\right)+m_{B(\mathrm{AB})}\cdot r_{A}\cdot \left(r_{B}-r_{\mathrm{AB}}\right)}$$

Here, *m*_a,113_ and *m*_a,115_ are the nuclide masses of indium isotopes, whereas *m*_A(AB)_ and *m*_B(AB)_ are the masses of indium from materials A and B that have been used to prepare mixture AB.

Although a single mixture of AB is sufficient to derive the isotope ratio correction factor, 14 mixtures were prepared with the resulting ^113^In/^115^In isotope ratios ranging from 0.06 to 12 (Table S2). This approach was undertaken to evaluate the reliability of the gravimetric isotope mixture method.

As shown in Figure 2, the isotope ratio correction factors obtained using eq 1 show a pronounced U-shaped dependence on the isotopic composition of the mixtures (*r*_AB_). This violates the underlying principle of the calibration model, which relies on the independence of the calibration factors from *r*_AB_. Similar behavior was observed by Lu et al. in their recent work on zinc isotopes.^15^ While the correction factors derived from mixtures AB7 and AB14 could be reasonably excluded due to their extreme isotopic compositions being too close to either material A or material B (AB7 contains A/B ≈ 100:1 and AB14 contains A/B ≈ 1:70), a persistent trend remains in the values of *K* at the middle (flat) part of the curve, which cannot be ignored. In their recent work, Lu et al. also observed “a linearly proportional variation in the middle (flat) part of the curves”.^15^ We have encountered similar considerations in our work on lead isotope ratio measurements^16^ and adopted the view that the binary mixtures ought to be made in such a way as to yield 1:1 isotope ratios. The approach of finding the isotope ratio factor corresponding to *r*_AB_ ≈ 1, however, remains unsatisfactory for indium, where samples of natural indium (*r* ≈ 0.043) are situated at the far-left part of the curve (Figure 2).

**Figure 2:**
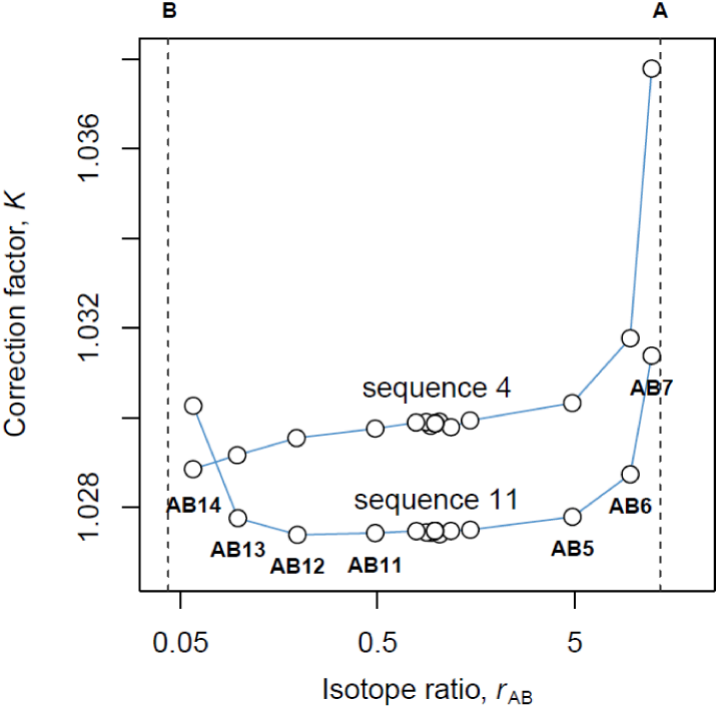
Indium isotope ratio (^113^In/^115^In) correction factors obtained using the classical single-mixture measurement model (eq 1) show a clear dependence on the isotopic composition of the mixtures (*r*_AB_) that contradicts the underlying calibration model. The compositions of all mixtures are given in Table S2. Dashed lines for A and B represent the ratios in pure indium-113 and pure indium-115 materials, respectively.

### A Revised Model for Isotope Ratio Calibration (Mixture Model)

An intuitive, yet arbitrary, approach to calibrate indium isotope ratios from natural samples would be to extrapolate the “flat part” of the curve (Figure 2) to *r*_AB_ ≈ 0.043. Instead, we propose that the U-shaped pattern seen in Figure 2 is due to somewhat biased isotope ratio measurements of pure materials A and B (Figure 3).

**Figure 3:**
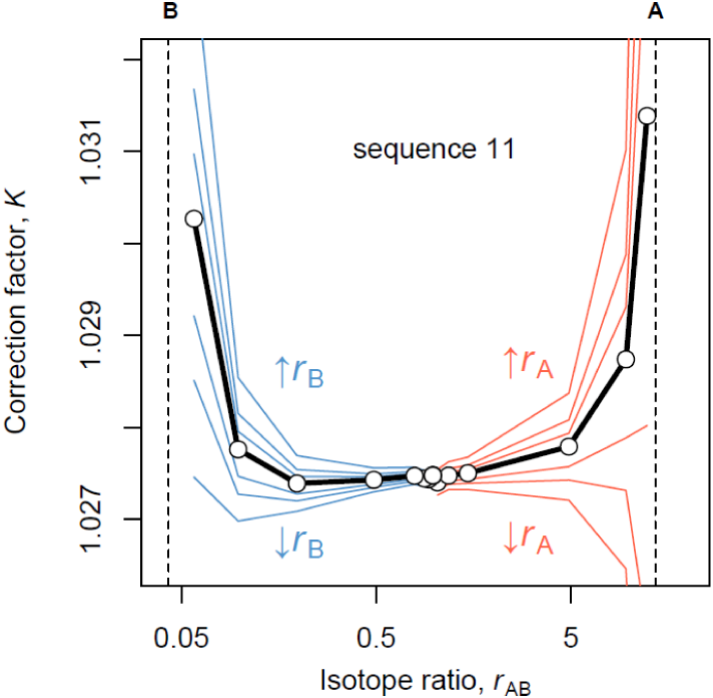
Systematic bias in the isotope ratio measurements of pure materials (A and B) causes the patterns observed in Figure 2 and by other studies, most notably Lu et al.^15^

The difficulties in obtaining the isotopic composition of pure isotopic spikes have long been recognized. It is, in fact, common practice to avoid introducing pure isotopic materials directly into the mass spectrometers altogether.^17^ This has led to the development of isotope dilution methods that rely on measuring the isotopic composition of mixtures of the primary standards and the isotopic spikes instead of pure isotopic materials.^18^ Following this tradition, we deliberately avoid using the measurements of pure materials A and B for the purposes of obtaining isotope ratio correction factors *K*.

A minimum of three mixtures (of A and B) are required to obtain *K* without relying on the direct measurements of *r*_A_ and *r*_B_.^11^ Although explicit measurement models are available to achieve this,^11^ numerical optimization methods were employed here instead of analytical expressions. In short, the values of *R*_A_, *R*_B_, and *K* are taken as model parameters. While conceptually this can be achieved, for example, by taking the measurement results from all 14 mixtures and finding the values of *R*_A_, *R*_B_, and *K* that minimize the dispersion between the observed and calculated isotope ratios, we have employed a more formal method of maximum likelihood to perform the model fitting.^19^ In other words, a single calibration factor is obtained for each measurement sequence by measuring only gravimetric mixtures, unlike the traditional single-mixture approach, which leads to a correction factor from each mixture and two pure materials (Figure S1). An example R code is provided in the Supporting Information although any software equipped with a minimization algorithm will suffice, including Excel.

In order to evaluate the effect of the maximum likelihood estimates (MLEs) of *R*_A_ and *R*_B_ on the values of the correction factors obtained by eq 1, the resulting MLEs of *R*_A_, *R*_B_, and *K* (for each measurement sequence) were used to substitute *r*_A_ and *r*_B_ (that is, *r*_A_ = *R*_A_/*K* and *r*_B_ = *R*_B_/*K*), which are required in eq 1. The resulting values of *K* from this hybrid method are shown in Figure 4 for each gravimetric mixture from measurement sequences Nr. 4 and 11.

**Figure 4:**
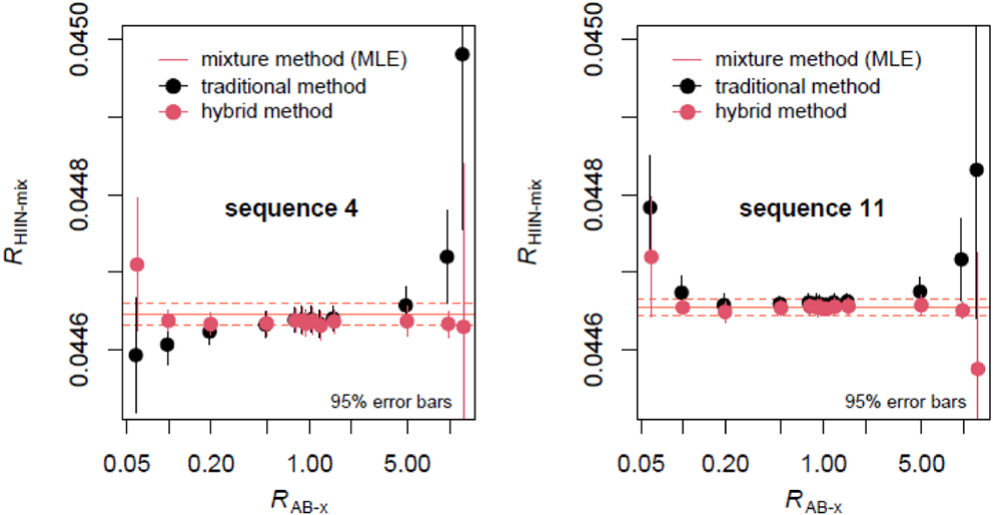
Comparison of the traditional method to obtain isotope ratio correction factors (eq 1) and a method that relies exclusively on measuring isotope mixtures (red line). The hybrid method also uses eq 1 but utilizes the values for *R*_A_ and *R*_B_ calculated from the mixture method to show the effect of these parameters on the resulting isotope ratios of indium in HIIN-mix.

It is evident that when the values of *r*_A_ and *r*_B_ obtained from the mixture model are used in eq 1, the resulting isotope ratio correction factors stabilize across the isotope mixtures. Figure 4 also shows that measurement biases in pure isotopic spikes become insignificant when instrumental isotopic fractionation is derived from a mixture having a 1:1 isotope ratio. Thus, traditional methods of data reduction (eq 1) can still be employed under such circumstances. Since the traditional and mixture models differ in their estimates of ^113^In/^115^In isotope ratios in materials A and B, we show them from all 16 measurement sequences in Figure 5. Thus, one can see that the traditional model overestimates both *R*_A_ and *R*_B_ from measurement sequence 11, which, in turn, leads to a U-shaped pattern shown in Figures 2 and 3.

**Figure 5:**
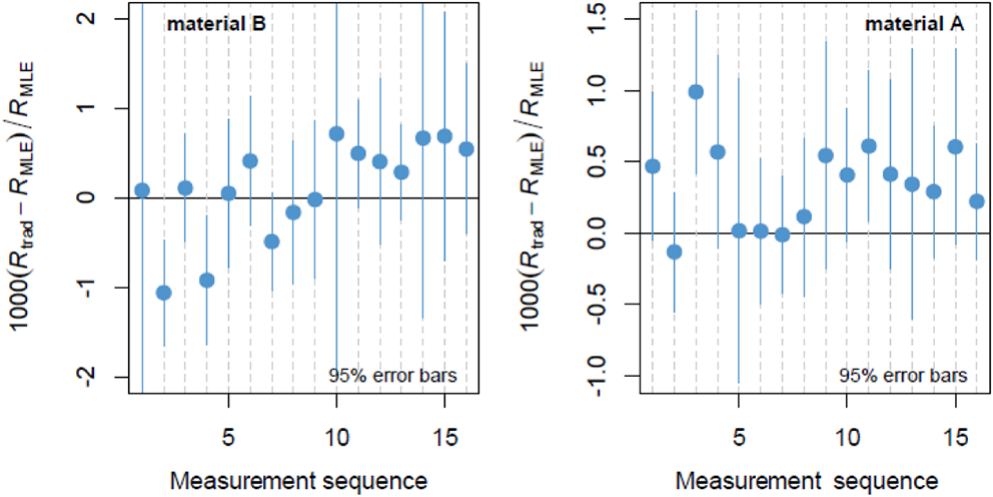
Relative difference of the estimated isotope ratios of indium in materials A and B using the traditional method (eq 1) and the mixture method.

Traditionally, isotope ratio calibrations have utilized gravimetric isotope mixtures with 1:1 isotope ratios, a practice that has also been widely adopted in quantitative analysis.^20^ However, this approach remains somewhat arbitrary, and little experimental evidence is available regarding the limitations of exact matching.^21^ Furthermore, when isotope ratio correction factors are found to exhibit a strong dependence on the isotope ratios of mixtures, it becomes unclear how to proceed. For example, two MC-ICP-MS studies on zinc isotope measurements have taken different approaches: one using the isotope ratio correction factors derived from isotope mixtures matching those of the samples,^15,22^ and another adopting the traditional approach by using the average of all correction factors.^23^ The mixture method used here not only sheds light on the cause of the observed isotope ratio correction factor patterns (Figure 3), but also provides a strong argument in favor of employing isotope mixtures with 1:1 isotope ratios in general (Figure 4).

### Data Analysis and Uncertainty Evaluation

In this work, we have adopted the mixture model over the traditional model and employed the maximum likelihood method to fit the measurement data to the model.^19,24^ We excluded mixtures AB7 and AB14 from the calculations owing to the fact that they are too close in their isotopic composition to materials A and B, respectively. The complete measurement model takes into account the following sources of uncertainty: (1) uncertainty associated with the chemical purity of indium in materials A and B, (2) uncertainty associated with the preparation of stock solutions A0 and B0, (3) uncertainty associated with the preparation of indium working solutions A1 and B1, (4) uncertainty associated with the preparation of gravimetric isotope mixtures of A1 and B1, (5) uncertainty associated with the isotope ratio measurements, including drift corrections, (6) uncertainty associated with the reproducibility of isotope ratio measurements between the measurement sequences, (7) uncertainty due to inhomogeneity of indium isotope ratios between the various units of HIIN-1, and (8) uncertainty associated with the atomic mass of the indium isotope.

#### Uncertainty Evaluation of Indium Stock Solutions

Chemical purity of indium in materials A and B was estimated by summing all impurities whose uncertainty is dominated largely by GDMS calibration. Elemental impurities that were below the detection limit were modeled with uniform distributions spanning from zero to the detection limit. From the data shown in Table S3, we obtain *w*(In, A) = 0.999 768(70) kg kg^–1^ and *w*(In, B) = 0.999 940(7) kg kg^–1^, where the values in parentheses are the standard uncertainties applicable to the last digits.

Mass fraction of indium in the stock solutions (A0 and B0) was obtained from the mass of indium metal and the mass of diluted solution while taking into account air buoyancy corrections, owing to the density difference between indium metal and its stock solution. The uncertainty associated with the resulting mass fractions was evaluated by using the Gauss method.

Working solutions (ca. 1 μg/kg) were prepared by serial dilution of stock solutions A0 and B0, involving three dilution steps. The uncertainty associated with the mass fraction of indium in the two sets of working solutions (A1-B2) was evaluated using the Monte Carlo method, recognizing that the estimates of indium mass fractions in solutions A1 and A2 are correlated, as they are both prepared from A0 (with the same considerations for B1 and B2). More details are provided in the Supporting Information.

#### Measurement Model

The uncertainties associated with the gravimetric isotope mixtures, repeatability of isotope ratio measurements, and drift corrections were propagated using a parametric statistical bootstrap.^19^ The measurement model contains two key stages. First, one establishes the true values for indium isotope ratios corresponding to each and every gravimetric mixture from the model parameters. The second step involves mapping these true isotope ratio values to the observed isotope ratios, which are biased by the instrumental isotopic fractionation and measurement drifts within each measurement sequence. Table 1 shows an outline of a simplified measurement model for a single measurement sequence, where no drift corrections are needed and where there is no uncertainty associated with gravimetric preparations or primary standards. The full annotated measurement model, written in R (a probabilistic programming language for statistical inference), is provided in the Supporting Information.

**Table 1:** Simplified Measurement Model

**Data**
*w*(In, A), *w*(In, B)
*m*_A_[1··· 14], *m*_B_[1··· 14]
mix_id[1··· *N*_obs_]
*r*[1··· *N*_obs_], *ur*[1··· *N*_obs_]
**Model parameters**
*R*_A_, *R*_B_, *K*
**Transformed model parameters**
*x*(^113^In, E) = *R*_*E*_/(1 + *R*_*E*_) where *E* = A, B
*x*(^115^In, *E*) = 1 – *x*(^113^In, *E*) where *E* = A, B
*M*(In, *E*) = *m*_a_ (^113^In) *x*(^113^In, *E*) + *m*_a_ (^115^In) *x*(^115^In, *E*) where *E* = A, B
*n*_z_,_*E*_ = *w*(In, E) *x*(^*z*^In, E)/*M*(In, *E*) where *z* = 113, 115 and *E* = A, B
*n*_true,*z*_[*i* = 1··· *N*_obs_] = *m*_A_[mix_id[*i*]] *n*_*z*,A_ + *m*_B_[mix_id[*i*]] *n*_*z*,B_ where *z* = 113, 115
*R*_true_[*i* = 1... *N*_obs_] = *n*_true,113_[*i*]/*n*_true,115_[*i*]
*r*_true_[*i* = 1... *N*_obs_] = *R*_true_[*i*]/*K*
**Likelihood**
*r*[*i* = 1... *N*_obs_] ∼ GAUSS(mean = *r*_true_[*i*], sd = *ur*[*i*])

### Isotopic Composition of Indium in HIIN-1

The ^113^In/^115^In isotope ratio in HIIN-1 and its associated uncertainty were obtained by pooling the results from individual estimates (shown in Figure 6) made over the course of 7 months. As a result, the associated combined uncertainty accounts for every aspect that reasonably contributes to the uncertainty of the measurement, including the homogeneity contribution in HIIN-1.

**Figure 6:**
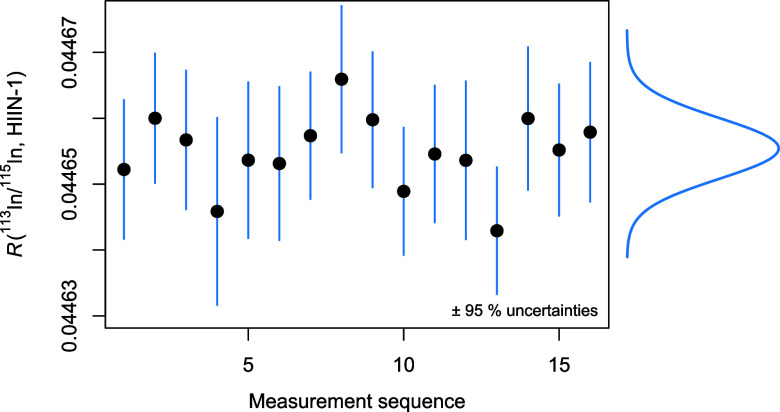
Measurements of ^113^In/^115^In indium isotope ratio in HIIN-1 over the course of 7 months. The probability density shown to the right represents the overall value, which includes the uncertainty associated with the sequence-to-sequence reproducibility.

The isotopic homogeneity of HIIN-1, evaluated for 15 HIIN-1 units against the mixture of the same 15 units over two measurement settings, was found to be δ­(^113^In) = −0.006 ‰, with a standard deviation of 0.021‰. The latter was taken as the estimate for the uncertainty associated with the isotopic homogeneity of HIIN-1. This source of uncertainty, albeit insignificant, was incorporated into the isotope ratio shown in Table 2.

**Table 2:** Isotopic Composition of Indium in the NRC HIIN-1 Isotopic CRM

quantity	value	expanded uncertainty, *k* = 2
Atomic weight, *A*_r_(In)	114.818 395	0.000 016
Isotopic abundance, *x*(^113^In)	0.042 746	0.000 008
Isotopic abundance, *x*(^115^In)	0.957 254	0.000 008
Isotope ratio, *n*(^113^In)/*n*(^115^In)	0.044 655	0.000 009
Isotope ratio, *n*(^115^In)/*n*(^113^In)	22.394	0.004
Isotope delta, δ_IPGP_(^113^In)	–0.049‰	0.033‰

Isotopic abundances and the corresponding atomic weight of indium in HIIN-1 were calculated as derived quantities from the ^113^In/^115^In isotope ratio using standard expressions relating these quantities.^25,26^ Nuclide masses and their covariances were taken from the 2020 Atomic Mass Evaluation report.^27^ The resulting isotopic composition is summarized in Table 2.

Isotopic composition of indium is often measured relative to a laboratory standard. For example, Liu et al. recently reported comprehensive MC-ICP-MS measurements of various terrestrial igneous rocks and geostandards using a Sigma-Aldrich indium solution (IPGP-In) as a bracketing standard.^9^ In order to connect these isotope delta measurements with our calibrated isotope ratio estimates in HIIN-1, we obtained the IPGP indium standard and performed isotope delta measurements of HIIN-1 relative to it using the aforementioned standard-sample bracketing method with an antimony internal standard (C-SSBIN).^12^ Our HIIN-1 indium was found to be slightly more depleted in ^113^In compared to the IPGP sample. The results are shown in Table 2, with additional details provided in the Supporting Information.

We find that nearly half of the uncertainty associated with the ^113^In/^115^In isotope ratio in HIIN-1 comes from the purity of material A, followed by isotope ratio measurements and drift corrections. The magnitudes of these components are provided in Table 3.

**Table 3:** Approximate Uncertainty Budget for the Indium Isotope Ratio in HIIN-1

uncertainty component	contribution to the combined uncertainty
Purity of material A (indium-113)	43%
Isotope ratio measurements (including drift corrections)	30%
Stock solution preparation	19%
Between-sequence reproducibility	18%
Gravimetric mixture preparation	5%
Purity of material B (indium-115)	3%
Isotopic homogeneity of HIIN-1	3%
Atomic mass of indium isotopes	<1%
Correlation between input quantities	–21%

### Comparison with Other Measurements

Indium isotope ratio measurements began in 1934 when Max Wehrli reported the discovery of the indium-113 isotope by observing ^115^In/^113^In ratio of 14:1 in optical spectra—a discovery prompted by the numerical value of the atomic weight of indium, which suggested the presence of a lighter isotope.^28^ Since then, many studies have been devoted to determining the isotopic composition of indium, with key literature summarized in Table 4.

**Table 4:** Published Mass Spectrometry Measurements of Indium Isotope Ratio

study, ref	*R*_113/115_	*u*(*R*_113/115_)	instrument^a^	calibration
Aston (1935)^30^	0.047	0.010	–	None
Blewett (1936)^31^	0.048	0.002	–	None
White (1948)^32^	0.0442	0.000 3	EIMS	Unknown
Hibbs (1949)^33^	0.0434	0.000 1	TIMS	Unknown
White (1956)^34^	0.04 526	0.000 44	TIMS or EIMS	Unknown
Saito (1987)^35^	0.04 498	0.000 02	EM-TIMS	Square-root mass correction
Saito (1987)^35^	0.04 495	0.000 12	FC-TIMS	None
Chang (1991)^36^	0.04 458	0.000 05	EM-TIMS	Square-root mass correction
Yang (2010)^7^	0.044 720	0.000 095	MC-ICP-MS	Regression method with SRM 978a silver
Shuai (2020)^8^	0.044 617	0.000 090^b^	MC-ICP-MS	Isobaric spike method with natural tin
This work	0.044 655 0	0.000 004 3	MC-ICP-MS	Gravimetric isotope mixture method with ^113^In and ^115^In

a EM: electron multiplier detector, FC: Faraday cup detector, MC: Faraday cup multicollector detector. EIMS: electron impact ionization mass spectrometry, TIMS: thermal ionization mass spectrometry.

b The reported combined uncertainty, *u*(*R*_113/115_) = 0.000 013/2, was revised to include the contribution from the calibrator, tin isotope ratio (^118^Sn/^120^Sn = 0.742 95), by summing the relative uncertainties in quadrature: [*u*(*R*_113/115_)/*R*_113/115_]^2^ = (0.5 × 0.000013/0.044617)^2^ + (0.2%)^2^. The relative uncertainty associated with the IUPAC recommended value for the ^118^Sn/^120^Sn ratio was estimated to be 0.2%.

Although no previous measurement can be considered fully calibrated—that is, believed to be free from instrumental isotopic fractionation effects—the International Union of Pure and Applied Chemistry (IUPAC) has recognized our previous measurement^7^ as the “best measurement” of indium isotopic composition from a single terrestrial sample.^29^ The indium sample utilized in our 2010 study (denoted S) and HIIN-1 have nearly identical isotopic compositions, giving an isotope delta value δ_HIIN‑1_(^113^In, S) = −0.278 ‰, with a standard deviation of 0.014‰, which allows direct comparison of these two measurements. Thus, the value reported in 2010, *R*(^113^In/^115^In, S) = 0.044 72 ± 0.00019 (95% confidence level), provides the corresponding value for HIIN-1,

R(In113/In115,HIIN‐1‐2010)=R(In113/In115,S)[1+δHIIN‐1(In113,S)]=0.044 73±0.000 19⁣(95 % confidence level)

which is nearly indistinguishable from *R*(^113^In/^115^In, S). Thus, our measurements from this study agree well with the previous value, based on the regression method with traceability to NIST SRM 978a silver isotopic reference material, while providing an order-of-magnitude improvement in measurement uncertainty.

A more recent work by Shuai et al. reported indium isotope ratio measurements using MC-ICP-MS, and a method that integrates the concepts of the double-spike and external normalization methods by measuring mixures of elements with at least one isobaric isotope (isobaric spike method).^8^ Similar to the double spike or regression methods, the isotope ratios of the spike element (in this case, tin from a NIST SRM 316a standard solution) need to be known beforehand, which is especially problematic in this case as there are no isotopic reference materials of tin and the natural isotopic composition of tin is poorly constrained. The IUPAC tabulated value for ^118^Sn/^120^Sn carries an associated relative uncertainty of ca. 0.2%, which we used to evaluate the uncertainty associated with the indium isotope ratio reported by Shuai et al. (Table 4).

The determinations of ^113^In/^115^In listed in Table 4 show remarkable and steady progress in isotope ratio measurements over time, achieving an average two orders of magnitude improvement in measurement uncertainty over the last century. While the dispersion between the results shown in Table 4 is nearly seven times larger than the reported uncertainties would suggest (Birge ratio, *R*_B_ = 6.6), this is likely due to the lack of calibration in the earlier results and a lack of comprehensive uncertainty evaluations. Nevertheless, good agreement among the four most recent studies suggests significant progress in determining the isotopic composition of indium.

### Standard Atomic Weight of Indium

Considering the last four determinations of the isotopic composition of indium (Table 4), we can establish a consensus value for the indium isotope ratio using the procedure of DerSimonian and Laird, as was done for the last several revisions of the standard atomic weights by IUPAC.^37^ Taking into account the isotope delta value between the materials HIIN-1 and IPGP-In, one obtains a consensus value *R*(^113^In/^115^In) = 0.044 656 for IPGP-In, having an associated standard uncertainty *u*(*R*) = 0.000 007.

There is scant literature on the natural variability of indium isotopes.^9,10^ Both studies use different reference standards but are connected by a common reference material, BHVO-2. Together, these two studies suggest a low value of δ_IPGP_(^115^In, JZN-1) = −0.06 ± 0.04 (2sd) and a high value of δ_IPGP_(^115^In, OU-3) = +0.65 ± 0.04 (2sd). This corresponds to an atomic weight of 114.8184 ± 0.0001 (±3s; see Supporting Information for calculations). Comparing this value to the current standard atomic weight of 114.818 ± 0.001 suggests that an additional decimal digit to the standard atomic weight of indium might be warranted.

## Conclusions

In this work, we report the first calibrated primary isotope ratio measurement of indium applied to the high-purity indium isotope reference material (HIIN-1). By employing an improved measurement model in the gravimetric isotope mixture method in combination with modern data analysis techniques, we achieved a relative uncertainty of 10 parts in 10^5^ for the indium isotope ratio—an order of magnitude improvement over our earlier measurements made in 2010. Furthermore, our results provide further validation of two recent indium isotope measurements that both relied on secondary isotopic standards and advance our understanding of the gravimetric isotope mixture method, which tnderpins high-precision isotope ratio measurements.

## Supplementary Material













## Notes

Supporting Information (PDF)
All measurement results of this study (XLSX)
Computational code, written in programming language R, used to analyze data and produce all figures and tables of this study (TXT)
Computational code, written in programming language R, to illustrate the calculation of model parameters (RA, RB, K) from a single measurement sequence using the method of maximum likelihood (TXT)
Data of the natural variations of indium isotope ratios and their least squares adjustment (XLSX)
Computational code, written in programming language R, used to calculate the atomic weight of indium (TXT)
