# Supplementary material for: Determination of the Isotopic Composition of Indium by MC-ICP-MS Using an Improved Measurement Model for the Gravimetric Isotope Mixture Method
Source: Anal Chem. 2025 Aug 15;97(33):17946–53. doi: 10.1021/acs.analchem.5c00878 (PMC12392257; doi:10.1021/acs.analchem.5c00878)
Supplement: Supplementary file 1 [file ac5c00878_si_001.pdf]

Supporting Information

Determination of the isotopic composition of indium by MC-ICP-MS using an improved measurement model for the gravimetric isotope mixture method

Juris Meija[1], Kenny Nadeau[1], Brad Methven[1], and Lu Yang[1]*

[1] National Research Council Canada, Metrology Research Centre, 1200 Montreal Rd, Ottawa, Ontario, K1A 0R6, Canada.

*Corresponding author: lu.yang@nrc-cnrc.gc.ca

Table of contents:

Table S1. MC-ICP-MS operating conditions

Table S2. Preparation of ^{113}In and ^{115}In gravimetric isotope mixtures for isotope ratio calibration

Table S3. Mass fraction of the elemental impurities (µg kg^{-1}) in the indium materials A and B, as measured by the GDMS

Figure S1. Comparison of the proposed multi-mixture approach (black lines), whereby a single calibration factor is obtained from each measurement sequence by measuring only gravimetric mixtures against the traditional single-mixture approach (red crosses) whereby each gravimetric mixture results a single correction factor. The individual correction factors can be found in FGIM-indium.xlsx file.

S4. Excel datafile (FGIM-indium.xlsx) containing all measurement results of this study

S5. Excel datafile (indium-natural-variations.xlsx) containing data about the natural variations of indium isotope ratios and their least squares adjustment

S6. Text file (indium-data-analysis.txt) containing computational code, written in programming language R, used to analyze data and produce all figures and tables of this manuscript.

S7. Text file (indium-MLE-R.txt) containing computational code, written in programming language R, to illustrate the calculation of model parameters (R_A, R_B, K) from a single measurement sequence using the method of maximum likelihood.

S8. Text file (indium-std-at-weight-R.txt) containing computational code, written in programming language R, to illustrate the calculation of proposed standard atomic weight of indium.

Table S1. MC-ICP-MS operating conditions

**Instrument settings**	
Radio-frequency power	1185 W
Plasma gas flow rate	16.0 L min^{-1}
Auxiliary gas flow rate	1.00 L min^{-1}
Sample gas flow rate	1.015 L min^{-1}
Sampler cone orifice (H, Ni)	1.1 mm
Skimmer cone orifice (Ni)	0.8 mm
Lens settings	Optimized for sensitivity and flat-top peak shape
**Data acquisition parameters**	
Faraday cup configurations	L3 (^{111}Cd), L1 (^{113}In), C (^{115}In), H2 (^{117}Sn);<br>L1 (^{113}In), C (^{115}In), H2 (^{121}Sb), H3 (^{123}Sb)
Mass resolution	low
Signal integration time	2.097 s
Numbers of integrations/cycles/blocks	1/10/4

Table S2. Preparation of ^{113}In and ^{115}In gravimetric isotope mixtures for isotope ratio calibration[a]

Sample	m_{A1}/g	m_{B1}/g	Sample	m_{A2}/g	m_{B2}/g
A1-1	15.04310	0	A1-2	15.06135	0
B1-1	0	15.06710	B1-2	0	15.07950
AB1-1	7.70115	7.34300	AB1-2	7.67840	7.35700
AB2-1	7.92040	7.13195	AB2-2	7.89820	7.11880
AB3-1	8.46115	6.54695	AB3-2	8.46930	6.57740
AB4-1	9.38220	5.62645	AB4-2	9.39830	5.62995
AB5-1	13.31915	1.70475	AB5-2	13.29140	1.69910
AB6-1	14.58380	0.43130	AB6-2	14.54490	0.43310
AB7-1	14.93615	0.10810	AB7-2	14.92630	0.11025
AB8-1	7.47790	7.55135	AB8-2	7.46085	7.54610
AB9-1	7.25540	7.77175	AB9-2	7.25590	7.76940
AB10-1	6.76000	8.27730	AB10-2	6.72970	8.21980
AB11-1	4.85010	10.15455	AB11-2	4.86300	10.21180
AB12-1	2.05545	12.94250	AB12-2	2.06585	12.87340
AB13-1	0.79885	14.24875	AB13-2	0.80950	14.26885
AB14-1	0.22425	14.83195	AB14-2	0.22425	14.78760

a. Mass fraction of indium in solutions A1 and A2 is w(In, A1) = 0.99901 mg kg^{-1} and w(In, A2) = 0.99904 mg kg^{-1}. Mass fraction of indium in solutions B1 and B2 is w(In, B1) = 0.99881 mg kg^{-1} and w(In, B2) = 0.99904 mg kg^{-1}. Additional details on the preparation of these solutions are provided in the supplementary information.

Table S3. Mass fraction of the elemental impurities (µg kg^{-1}) in the indium materials A and B, as measured by the GDMS

Element	Material A	Material B	Element	Material A	Material B
Li	<0.2	0.1	Pd	38	1
Be	<0.2	0.1	Ag	66	6500
B	6	0.15	Cd	<180	14000
C	490	97	In	matrix	matrix
N	13	4	Sn	2560	22900
O	380	190	Sb	17100	120
F	<7	1	Te	80	8
Na	2	5	I	<2	1
Mg	4	0.35	Cs	<0.3	0.3
Al	19	10	Ba	<2	0.45
Si	180	76	La	<0.08	0.035
P	5	0.35	Ce	<0.1	0.1
S	8540	17	Pr	<0.09	0.04
Cl	<4	33	Nd	<0.3	0.15
K	<110	16	Sm	<1	0.2
Ca	<8	23	Eu	<1	0.5
Sc	<0.2	0.05	Gd	<0.3	0.15
Ti	0.7	0.15	Tb	<0.08	0.035
V	<0.2	0.1	Dy	<0.3	0.15
Cr	<0.5	10	Ho	<0.08	0.035
Mn	<0.3	3	Er	<0.2	0.1
Fe	590	260	Tm	<0.1	0.035
Co	12	2	Yb	<0.2	0.1
Ni	1370	740	Lu	<0.08	0.035
Cu	110000	340	Hf	<0.9	0.25
Zn	950	32	Ta	<1740	165
Ga	10	0.5	W	<0.7	0.3
Ge	<8	1	Re	<0.7	0.3
As	64	80	Os	<4	1
Se	<6	2.5	Ir	<2	0.35
Br	<24	7.5	Pt	55500	0.45
Rb	<0.4	0.15	Au	<190	16.5
Sr	<0.1	0.05	Hg	<9	4
Y	<0.2	0.05	Tl	150	62
Zr	<0.2	0.1	Pb	31800	14400
Nb	<0.4	0.035	Bi	7	340
Mo	<1	0.5	Th	0.4	0.1
Ru	<0.8	0.35	U	4	0.045
Rh	<1620	0.25			

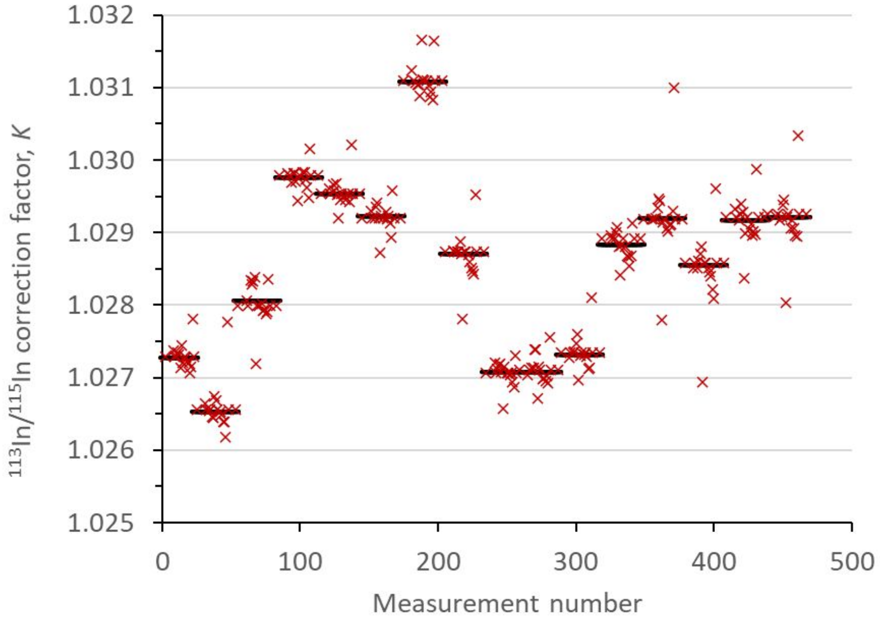

Figure S1. Comparison of the proposed multi-mixture approach (black lines), whereby a single calibration factor is obtained from each measurement sequence by measuring only gravimetric mixtures against the traditional single-mixture approach (red crosses) whereby each gravimetric mixture results a single correction factor. The individual correction factors can be found in FGIM-indium.xlsx file.
